# The Cross-Talk Between the TNF-α and RASSF-Hippo Signalling Pathways

**DOI:** 10.3390/ijms20092346

**Published:** 2019-05-11

**Authors:** Delvac Oceandy, Bella Amanda, Faisal Yusuf Ashari, Zakiyatul Faizah, M Aminudin Aziz, Nicholas Stafford

**Affiliations:** 1 Division of Cardiovascular Sciences, Faculty of Biology, Medicine and Health, University of Manchester, Manchester M13 9PT, UK; nicholas.stafford@manchester.ac.uk; 2 Department of Biomedical Science, Faculty of Medicine, Universitas Airlangga, Surabaya 60132, Indonesia; bellaamanda1704@yahoo.com (B.A.); faisalyusufashari@gmail.com (F.Y.A.); faizbiomed@yahoo.com (Z.F.); azizandro@gmail.com (M.A.A.)

**Keywords:** TNF-α, Hippo pathway, RASSF, apoptosis, signalling pathway

## Abstract

The regulation of cell death through apoptosis is essential to a number of physiological processes. Defective apoptosis regulation is associated with many abnormalities including anomalies in organ development, altered immune response and the development of cancer. Several signalling pathways are known to regulate apoptosis including the Tumour Necrosis Factor-α (TNF-α) and Hippo signalling pathways. In this paper we review the cross-talk between the TNF-α pathway and the Hippo signalling pathway. Several molecules that tightly regulate the Hippo pathway, such as members of the Ras-association domain family member (RASSF) family proteins, interact and modulate some key proteins within the TNF-α pathway. Meanwhile, TNF-α stimulation also affects the expression and activation of core components of the Hippo pathway. This implies the crucial role of signal integration between these two major pathways in regulating apoptosis.

## 1. Introduction

In a complex biological system, it is essential that the number and the type of living cells in each organ are maintained at precise levels. Therefore, it is equally important to remove unwanted cells by programmed cell death, or apoptosis, to keep the desired cell population alive. For example, during organ development apoptosis is essential in controlling organ size/cell numbers, shaping organ structure, eliminating dysfunctional cells and removing unwanted structures [1]. Defective apoptosis may lead to major anomalies in organ development. Other than during embryonic development, apoptosis is also key in a number of physiological processes such as the maturation and development of the immune response and nervous system [2,3].

Thus, it is understandable that any abnormality in the regulation of apoptosis may cause many types of disease. For example, enhanced apoptosis is a feature of neurodegenerative diseases, myocardial infarction, heart failure and some types of autoimmune disease [4,5]. In contrast, decreased apoptosis may be associated with cancer and some autoimmune disorders [6,7]. 

Therefore, precise regulation of apoptosis at the molecular and cellular level is crucial to organ homeostasis. In essence, the molecular control of apoptosis can be classified in two distinct pathways: the extrinsic and the intrinsic pathways. The fundamental difference between these two pathways is the source of the initial inducers. The extrinsic pathway is induced via stimulation of membrane receptors by extrinsic signals, for example TNF-α, Fas ligand and TNF-related apoptosis-inducing ligand (TRAIL). Meanwhile, the intrinsic pathway is initiated by internal stimuli, such as DNA damage, hypoxia and metabolic stress, that eventually increase mitochondrial membrane permeability [8].

However, one of the most important aspects of apoptosis regulation is the mechanism behind the transmission and translation of the initial inducer signal into the activation of the common pathways of the apoptotic machinery. The extrinsic pathway starts with the binding of apoptotic ligands to their receptors, which induces formation of death inducing signalling complex (DISC), leading to activation of caspase 8 and eventually activation of the effector caspases (caspase 3/7). The intrinsic pathway is slightly more complicated, involving members of the Bcl-2 family of proteins. Activation of this pathway leads to the release of cytochrome c due to increased mitochondrial membrane permeability. Cytochrome c interacts with apoptosis protease-activating factor 1 (Apaf-1), and forms a complex known as the apoptosome, which in turn activates the effector caspases [8].

As with many other molecular signalling pathways, the presence of negative regulatory systems that counteract and balance the signal is very important. For example, there is balance between the pro-inflammatory and the anti-inflammatory cytokines, balance between the kinases and the phosphatases, and balance between the oncogenes and tumour suppressor genes, which are all very important in maintaining cell homeostasis. Indeed, in the case of apoptosis the presence of anti-apoptotic pathways is also essential. There are several anti-apoptotic molecules that have been very well characterised such as extracellular signal–regulated kinases 1 and 2 (ERK1/2), Akt, β-catenin and the Yes-associated protein-Tafazzin/TEA domain family member (YAP-TAZ/TEAD) complex which is closely related to the Hippo signalling pathway.

The Hippo signalling pathway has attracted a lot of attention in recent years. The strong involvement of this pathway in regulating both cell survival and cell death/apoptosis is of particular importance in several different pathophysiological processes, for example in tumour formation, regeneration and inflammation [9]. The core components of this pathway comprise of kinases Mammalian Ste20-like kinase 1 (MST1), MST2, Large tumour suppressor 1 (LATS1) and LATS2 as well as adaptor molecules Salvador homolog (SAV1) and MOB kinase activator 1 (MOB1). When active, the Hippo pathway suppresses the YAP-TAZ/TEAD complex and is therefore pro-apoptotic. Interestingly, components of this pathway interact with many other regulators of cell apoptosis, notably members of the TNF-α pathway. Cross-talk between major signalling pathways is known as an important aspect in the control of many cellular functions. Thus, in this paper we will review some recent findings that describe the interaction and crosstalk between members of the Hippo pathway and the TNF-α signalling components. 

## 2. The TNF-α Pathway

### 2.1. TNF-α and TNF Receptor Complex

TNF-α is known as a pro-inflammatory cytokine, mainly produced by inflammatory cells such as monocytes, macrophages and activated T-cells, along with other cell types including endothelial cells and fibroblasts. The precursor protein of TNF-α (pro-TNF-α or membrane TNF-α) is a transmembrane protein that requires TNF-α converting enzyme (TACE) to be converted into soluble TNF-α [10]. 

Both types of TNF-α exert their function by binding to TNF-receptor (TNFR). There are two types of TNF-α receptors, TNFR1 and TNFR2. TNFR 1 can be activated either by transmembrane TNF or soluble TNF, while TNFR2 is activated primarily by soluble TNF [11]. Both TNFRs consist of an extracellular domain consisting of 182 amino acids, a transmembrane domain consisting of 21 amino acids, and an intracellular domain consisting of 221 amino acids. Both TNFR1 and TNFR2 have four cysteine-rich regions located in the extracellular domain; however, there are differences between the two TNF receptors. One such difference is the death domain (DD), a homophilic interaction region of approximately 80 amino acid residues, which is present in the intracellular domain of TNFR 1 but absent in TNFR2. The DD is involved in transmitting apoptosis signals, the antiviral response, and induction of nitric oxide synthesis [11,12].

Binding of TNF-α to the receptors leads to homotrimerization of the TNFR subunit [10]. The death domains combine to form a structure needed to promote interaction with the death domain of TNFR-associated death domain protein (TRADD). This induces recruitment of several signalling molecules such as TNFR-associated factor (TRAF), Receptor-interacting serine/threonine-protein kinase 1 (RIPK1) and Fas associated protein with death domain (FADD), to the receptor complex [12]. This complex activates a number of downstream signalling pathways, of which the best known are the apoptosis signalling and the Nuclear factor kappa-light-chain-enhancer of activated B cells (NF-κB) pathways. Other downstream effectors include the ubiquitin-mediated proteolysis and TNF-induced pro-inflammatory pathways [10,12].

### 2.2. TNF-α Dependent Apoptosis Pathway

The TNF-α induced apoptosis pathway is well characterized. It is mainly modulated through signalling by TNFR1. Activation of this pathway involves the recruitment of molecules to form the “death inducing signaling complex” (DISC). Components of this complex include TRADD, FADD and TRAF1 [10]. Formation of DISC also requires the release of silencer of death domain (SODD) [13]. DISC recruits and activates initiator caspases such as caspase 8. Active caspase 8 initiates activation of executioner caspases (caspase 3 and caspase 7). Caspase 3 is the main downstream effector of the TNF-α induced apoptosis since it is responsible for genomic DNA degradation via activation of DNAse which essentially causes cell death [10]. Although TNF-α is a strong inducer of apoptotic cell death, it is important to note that it acts mainly in abnormal cells such as tumour cells or virally infected cells.

### 2.3. Regulation of NF-κB Pathway

In contrast to its role in the induction of apoptosis pathways, TNF-α may also induce cell survival via activation of the NF-κB [12]. In addition, activated TRAF2 and TRADD may stimulate not only the NF-κB pathway but also the mitogen activated protein kinase (MAPK) and the c-Jun-N-terminal kinase (JNK) pathways [14]. In an inactive state NF-κB binds to its inhibitory subunit IκB. Upon activation by TNF-signalling, TRAF2 and receptor interacting protein kinase (RIP) activate the inhibitor of NF-κB kinase (IKK), which in turn phosphorylate and release IκB from the NF-κB complex. The activated NF-κB is then translocated to the nucleus and stimulates gene transcription [10]. On the other hand TRAF2 also interacts with members of the MAP kinases, and this can also result in the activation of this pathway [15]. The activation of NF-κB as well as the MAPK pathway by TNF-α may produce different effects depending on the cell type. NF-κB activation in inflammatory cells mainly produces a pro-inflammatory response and secretion of pro-inflammatory cytokines; however, in cells such as cardiomyocytes activation of NF-κB by TNF-α is associated with increases in intracellular calcium due to the activation of calcium handling molecules [16]. 

### 2.4. TNF-α Mediated Inflammatory Pathway

It is known that TNF-α has an important role in the inflammatory process. TNF-induced inflammation mainly occurs via the NF-κB and MAPK signalling pathways. However, there are several alternative pathways through which TNF-α can initiate inflammatory response. These include the induction of the sphingomyelinase pathway, which leads to the generation of diacyl glycerol and protein kinase C and eventually results in the activation of NF-κB via an alternative pathway [10]. In addition, TNF-α also activates 5-lipoxygengase and phospholipase A2 enzymes, leading to the production of arachindonic acid, 5-hydroxyeicosatetraenoic acid (5-HETE) and proinflammatory leukotrienes [17].

Overall, TNF-α signalling appears to have pleiotropic roles including regulation of cell death/apoptosis, the immune response, cell survival/proliferation and protein ubiquitination. Functions of TNF-α are likely to be different depending on the cell types. It is also apparent that diverse interaction partners between components of TNF-α signalling and other major signalling molecules determine the downstream effects of the signal. One major signalling pathway to have recently come to the fore in the area of cell death, survival and proliferation is the Hippo signalling pathway. In the following section we will focus on reviewing recent findings that link the Hippo pathway and TNF-α signalling. 

## 3. The Hippo signalling Pathway

### 3.1. Core Components of Hippo Pathway

The Hippo pathway was first discovered in *Drosophila*, with the characterization of hippo, salvador (sav) and warts via a genetic mosaic screen for potential tumor suppressor and oncogenes [18]. When these genes were mutated, the *Drosophila* exhibited a “Hippo-like appearance” due to organ overgrowth [19]. Hippo is a kinase which forms a complex with adaptor molecules sav to activate warts. Further downstream warts phosphorylates, and hence deactivates transcriptional co-activator yorkie (Yki), and the growth control by the Hippo pathway acts principally through the inhibition of this molecule. It has been shown that Yki overexpression produces a phenotype which resembles that of loss-of-function mutations in hippo, sav, warts and mats [20]. The pathway has now been relatively well characterized from cell membrane to cytosol and nucleus [9]. 

**Figure 1:**
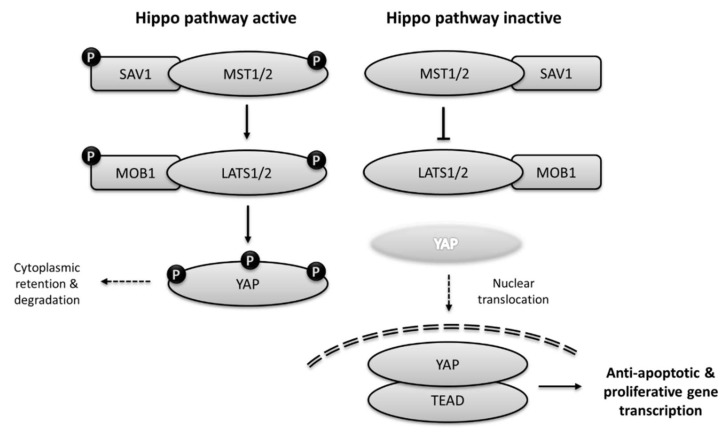
The core components of the mammalian Hippo signalling pathway. When active (left) Mammalian Ste20-like kinases (MST kinases) phosphorylate Salvador (SAV1) and downstream MOB kinase activator 1 (MOB1) and Large tumour suppressor (LATS) kinases, leading to phosphorylation and degradation of Yes-associated protein (YAP). When upstream kinases are inactive (right), non-phosphorylated YAP resides in the nucleus and binds to transcription factors to promote pro-survival and anti-apoptotic gene transcription.

The Hippo pathway is highly conserved among metazoans. The components of the mammalian Hippo pathway are functionally very similar to their *Drosophila* ortholog [21]. The mammalian orthologs of Hippo, Mammalian STE-20-like kinases 1 and 2 (MST1/2) are activated by auto-phosphorylation following dimerization [22]. However, MST1/2 may also be phosphorylated by other upstream kinases such as TAO1 [23]. Phosphorylated MST1/2 forms a complex with SAV1 (salvador ortholog) and phosphorylates the serine residues in its linker region, as well as phosphorylating another scaffolding molecule MOB1, which helps to recruit LATS1/2 (warts orthologs). Following recruitment to the complex, LATS1/2 are then phosphorylated by MST1/2 leading to their activation and phosphorylation of their main target, the Yes-associated protein (YAP) [24]. YAP is an ortholog of yorkie, and phosphorylation of this molecule leads to its inactivation, cytoplasmic retention and protein degradation. When the Hippo pathway is inactive, non-phosphorylated YAP is translocated to the nucleus and binds to transcription factor TEAD to promote expression of genes involved in cell proliferation and inhibition of apoptosis (Figure 1) [9,21,24].

### 3.2. Upstream Regulators of the Hippo Pathway

There are a number of different pathways which may regulate YAP, either through the core Hippo kinase cascade or independent of the central Hippo components. These include regulation by extracellular signals, for example via the extra cellular matrix, cell to cell contact, G-protein coupled receptor (GPCR) signalling and cell polarity [9]. 

The maintenance of cell polarity is important to sustain cell function. For example, epithelial cells normally attach to their neighbouring cells via complexes called adherens junctions (AJs), desmosomes, and tight junctions (TJs). The presence of TJs and AJs divide the plasma membrane into apical and basolateral domains and hence establish apical-basal polarity. Interestingly, proteins that are important in building or maintaining apical-basal polarity have been shown to modulate the Hippo pathway. These include: E-cadherin, which causes YAP inactivation [25]; Ajuba, which can interact with SAV1 and LATS1/2 kinases and displays inhibitory effects on YAP [26]; and LKB1 (liver kinase B1), which is capable of inducing phosphorylation of YAP [27]. Other molecules that are involved in apical-basal polarity and linked with the Hippo pathway include: NPHP4 (nephronophthisis 4), which can interact with and inhibit LATS1 [28]; ZO-2 (zone occludens-2), which can induce YAP nuclear localization [29]; and ZO-1 that has been shown to suppress TAZ activation [30]. Angiomotin (AMOT) and angiomotin-like 1 can also mediate YAP cytoplasmic retention [31,32]. In essence, the presence of structures that define cell polarity can lead to the induction of pathways that result in YAP inactivation, and thereby reduce proliferation and growth. 

Extracellular matrix components and tension have recently been regarded as important modulators of major signalling pathways. In the case of the Hippo pathway, extracellular matrix (ECM) components seem to be an important factor as well. For example Agrin, a unique component of the embryonic ECM, has recently been identified as a strong modulator of YAP activity and can thereby induce cell growth and proliferation [33,34,35].

An additional group of extracellular factors important for Hippo signalling are GPCR ligands such as the LPA (lysophosphatidic acid). LPA was identified as a positive modulator of YAP via binding and activation of the Gα12/13-coupled receptor, and was associated with inhibition of LATS1/2 [36]. This finding opens up the possibility to modulate the Hippo pathway by stimulation of membrane receptors. 

### 3.3. Roles of Hippo Pathway in Mediating Cellular Processes and Organ Functions

The physiological functions of the Hippo pathway are determined by the cellular function of its core components (for example MST1/2 and LATS1/2) and the main downstream effector YAP. The Hippo core components act as negative regulators of YAP, but also act independently of YAP to promote apoptosis [37,38]. Meanwhile YAP, if activated, has been known to regulate many aspects of cellular processes including cell proliferation, growth and maintenance of cell stemness, as well as influencing cell fate and differentiation [9]. Thus, it is understood that the physiological functions of the Hippo pathway range from controlling organ size during organogenesis and the development of cancers, to promoting tissue regeneration and repair as well as the maintenance of progenitor and stem cells.

The control of organ size by the Hippo pathway has been demonstrated in mice with genetic knockout of MST1 and MST2, which display profound liver overgrowth [39]. In addition, YAP is involved in kidney and urinary tract development [40]. In terms of the role in promoting tissue regeneration and repair, the primary example comes from the cardiac field. Several studies have shown that through genetic or pharmacological activation of YAP [41,42], or alternatively via inhibition of core Hippo components (SAV1) [43], heart regeneration can be induced following myocardial infarction. However, modulation of the Hippo pathway is somewhat of a double-edged sword, because activation of YAP or inhibition of core Hippo components are also strongly associated with cancer development. For example, studies using mouse models have demonstrated that double knockout of MST1 and MST2 might result in the development of liver cancer, colon cancer and lymphoblastic leukemia [44,45,46]. Likewise, a LATS1/2 have been associated with cancer cell growth, suppression of cancer immunity and breast cancer progression [37,47,48]. The involvement of LATS1/2 in ovarian tumours has also been reported [49]. Meanwhile, YAP overactivation is implicated in liver cancer as well as pancreatic and squamous cell carcinoma [50,51,52]. Although few mutations in Hippo pathway genes have been associated with human cancers, there are a number of studies that report the dysregulation of the Hippo pathway components in many types of human cancers such as liver, lung, colon, ovarian and prostate cancers [53].

### 3.4. RASSF Family Proteins as Major Upstream Regulators of Hippo Pathway

**Figure 2:**
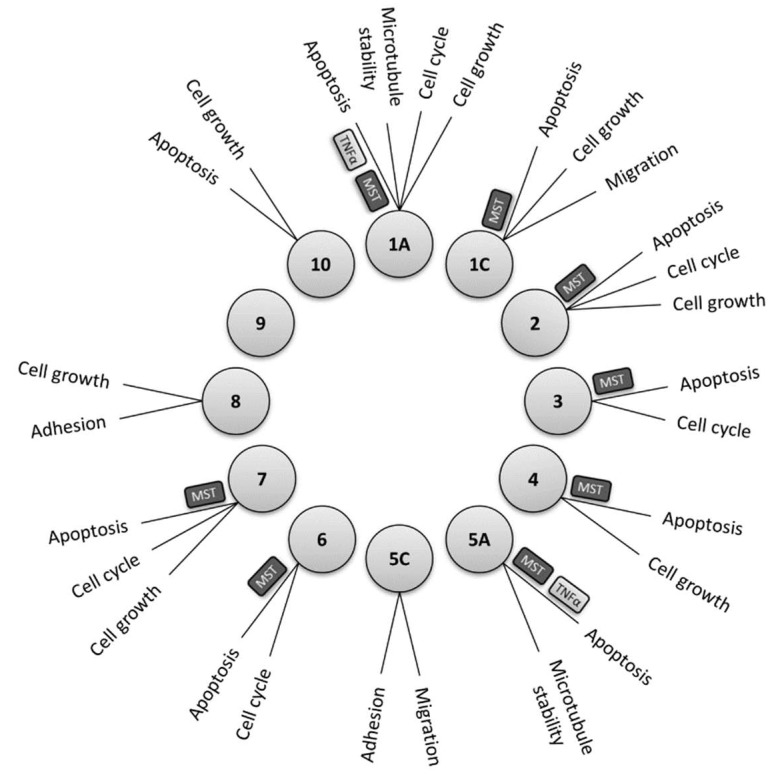
The major known cellular processes governed by each of the RASSF family proteins. Links to the Hippo (MST) and TNF-α signalling pathways are shown for relevant isoforms.

The Ras-association domain family member (RASSF) family proteins consist of ten members (RASSF1-10), several of which are understood to regulate and interact with components of the Hippo pathway. All members of this group of proteins are known to have a Ras-association domain, which can be located either in the N-terminus, as is the case in RASSF 7–10, or in the C-terminus as seen in RASSF 1–6 [54,55]. A number of studies have revealed that some RASSF proteins have additional characteristic domains enabling them to function as adaptor proteins. These include a protein kinase C (PKC) conserved region C1 domain in RASSF1A and 5A, an ataxia telangiectasia mutated (ATM) domain containing a phosphorylation motif in RASSF1A & 1C, and a C-terminally located Salvador RASSF Hippo (SARAH) domain in RASSF1–6 [55]. These structural differences may account for the variety of interacting partners amongst family members, and their diverse roles in regulating cellular functions such as growth, cell cycle regulation, microtubule stability, apoptosis, migration and adhesion as depicted in Figure 2.

The SARAH domain largely mediates interaction between RASSF proteins and members of the Hippo signalling pathway, in particular MST1 and MST2. Of the ten members of the family it is understood that RASSF1A, RASSF1C, RASSF2, RASSF3, RASSF4, RASSF5A (Nore1A), RASSF6 and RASSF7 show interactions with either MST1, MST2 or with both [56]. In addition, recent findings suggest involvement of RASSF proteins in mediating other important pathways such as the NF-κB and Wnt signalling pathways [57].

As a consequence, RASSF family proteins have been implicated with a number of important cellular processes and organ function. Since the canonical Ras-Raf-Mek-MAPK pathway regulates growth and development, RASSF1A, which is closely linked to this pathway, is involved in the regulation of cell growth, proliferation and oncogenesis. RASSF1A has been shown to be a strong modulator of cardiac growth (hypertrophy), whilst the involvement of this molecule in cancer and tumorigenesis is also evident [56,58].

Although RASSF family protein members mainly interact with MST1/2 via a similar pattern, i.e., mediated by the SARAH domain, the implications of the interaction may be variable. RASSF1A and RASSF5 are known to positively modulate MST-kinase activity [59], whereas one study has indicated a possible inhibitory effect of RASSF6 upon MST2 activity [60]. Nevertheless, interaction with MST1 and MST2 may account for the crucial function of RASSF proteins in mediating apoptosis. RASSF5A and RASSF1 are required to mediate the pro-apoptotic activity of MST kinases, thus it is understandable that the loss of these molecules, either due to mutations or epigenetic modifications, is strongly associated with development of cancer.

As discussed above, both the TNF-α and the Hippo signalling pathway together with the associated RASSF family proteins, are crucial in the regulation of cell apoptosis. As cross-talk between signalling molecules is essential in achieving balance between pro- and anti- apoptotic signals, it is very important to understand if the TNF-α pathway is also linked with the Hippo-RASSF pathway. Several studies have reported indications of such interactions which will be outlined below. 

## 4. Regulation of TNF-α Signalling by RASSF1A

RASSF family members have been linked to death receptor signalling through interactions with modulator of apoptosis 1 (MOAP1) [61,62]. Apoptotic signals can be activated by binding of the extracellular apoptotic mediators such as Fas ligand, TNF-α or TRAIL to their membrane bound receptors (Fas, TNF-R1 and TRAIL-R). This leads to the formation of DISC and activation of the caspase signalling cascade as mentioned above. An alternative crosstalk between intrinsic and extrinsic pathways may occur through caspase 8-mediated activation of executioner caspases 3 by Bax and cytochrome c, which is released from the mitochondria due to mitochondrial outer membrane permeabilisation [8]. This latter pathway is where RASSF and MOAP1 enter the picture. RASSF1A can bind to the MOAP1/TNF-R1 complex to facilitate an interaction between MOAP and Bax, thus driving Bax activation and MOMP to induce apoptosis [61]. 

The protein kinase C conserved region 1 (C1) domain at the N-terminal region of RASSF1A is essential for its association with the TNF-R1/MOAP1 and TRAIL/MOAP1 complexes [61]. Furthermore, a motif within the SARAH domain is known to mediate the interaction between RASSF1A and MOAP1 from the death pathway [61,62]. Recent studies have also revealed RASSF3 and RASSF6 to be MOAP1 binding partners, possibly mediated via the same motif [63,64]. These interactions indicate the general role of the RASSF family in pro-apoptotic regulation, with increasing evidence of their involvement in both the Hippo and the death receptor apoptotic pathways.

An interaction between RASSF1A and the components of the TNF receptor complex has also been observed in cardiomyocytes [16]. Interestingly, this interaction is implicated in the regulation of intracellular calcium homeostasis through modulation of phospholipase A2 (cPLA2), which in turn promotes activation of calcium transporters via protein kinase A. RASSF1A has been shown to positively mediates the inotropic effects of TNF-α on cardiomyocyte contraction [16]. These findings demonstrate variable effects of RASSF1 and TNF receptor signalling on cellular function, dependent upon the type of cells in which it takes place.

RASSF1A is also implicated in the NF-κB signalling pathway. It inhibits signal transmission from the proximal complex involving toll-like receptor/ Myeloid differentiation primary response 88 (TLR/MyD88), TRAF6 and Interleukin-1 receptor-associated kinase (IRAK) [65]. The outcome is the downregulation of the NF-κB mediated inflammatory response as shown in a model of acute intestinal inflammation [65]. Interestingly, in cardiac fibroblasts RASSF1, via modulation of MST1, inhibited TNF-α production via repression of the NF-κB pathway [66]. This may also explain the anti-hypertrophic nature of RASSF1A in the heart.

Overall, the interaction between members of the RASSF family proteins and components of TNF-α signalling is evident. The physiological implications are varied depending on cell type and the organ. 

## 5. Intersection between TNF-α Signalling and the Hippo Pathway

### 5.1. Modulation of LATS2 by TNF-α 

LATS2 is one of the central components of the Hippo pathway. It functions as a tumour suppressor and thus loss of LATS2 function is associated with many types of tumour such as soft tissue sarcomas, leukaemia and breast cancer [37]. In addition to negatively regulating YAP by phosphorylation, LATS2 also regulates cell proliferation through modulation of cell cycle regulators [67] and promotes apoptosis via downregulateion of B-cell lymphoma 2 (Bcl-2) and B-cell lymphoma-extra large (Bcl-xL) [68]. On the other hand LATS2 expression may also be regulated by many different factors. For example, p53 can induce LATS2 expression [69]. In contrast, some micro RNAs (miR-31, miR-372 and miR-373) are known to inhibit LATS2 expression [37].

Recently, Dong et al. have demonstrated regulation of LATS2 expression by TNF-α. By using an oral squamous cell carcinoma cell line (HN6 cells) they discovered that treatment with TNF-α not only induced LATS2 expression but also induced YAP phosphorylation and hence reduced its co-transcriptional activation [70]. The induction was dependent on both dose and time of TNF-α stimulation. We would expect the net effect of TNF-α treatment to therefore enhance the pro-apoptotic and anti-proliferative nature of LATS2. Hence this study potentially reveals a novel mechanism for the pro-apoptotic effect of TNF-α, in addition to the established mechanisms discussed above. 

### 5.2. Modulation of YAP-TEAD Activity by TNF-α Cancer Cells and Chondrocytes

Similar to its effects on LATS2 expression, TNF-α is also reported to induce YAP expression. Using a breast cancer cell line, Gao et al. observed that treatment with TNF-α or macrophage conditioned medium induces the expression level of YAP [71]. Interestingly, they also linked the regulation of YAP to the TNF-α-induced NF-κB activation. Following the binding of TNF-α to its receptor, activated IKKβ/ε might stimulate phosphorylation of YAP in the cytoplasm. This might induce the formation of a complex consisting of phosphorylated-YAP, TEAD and the p65 sub-unit of NF-κB to the nucleus to enhance gene transcription [71].

Although this is an interesting finding, it is contrary to the accepted view that YAP phosphorylation normally leads to its cytoplasmic retention and hence inactivation [9,24]. It is not clear whether in MCF7 cells active IKKβ/ε phosphorylates YAP at a different residue than those commonly known to cause YAP inactivation. Nevertheless, Gao and colleagues demonstrated that the impact of this cross talk was to enhance cell migration due to induction of HK2 expression [71]. Cell migration is indeed an important aspect during carcinogenesis and metastasis of cancer cells, therefore this finding adds to growing evidence of the potential significance of TNF-α/Hippo signal crosstalk.

In contrast to these findings in breast cancer cells, studies have also shown that TNF-α can inhibit YAP. Using the A172 glioblastoma cell line, Lu et al. found TNF-α treatment to dose-dependently promote apoptosis via increased mitochondrial fission and dysfunction. This coincided with a reduced expression of YAP, whilst YAP overexpression was shown to prevent the TNF-α induced apoptosis and mitochondrial dysfunction [72].

Similar findings have also been observed in chondrocytes. Deng and colleagues showed robust YAP expression in young mouse cartilage, which deteriorated with age and upon surgical induction of osteoarthritis. They went on to show that treatment of primary chondrocytes with TNF-α led to degradation of YAP and TAZ via TAK1-mediated phosphorylation, independently of upstream members of the Hippo pathway. Meanwhile, YAP overexpression inhibited TNF-α induced JNK and NF-κB signalling activation, and could thus provide a target to prevent cartilage degradation in osteoarthritis [73]. 

### 5.3. TNF-α Promotes YAP Activation in Endothelial Cells 

The most recent evidence describing TNF-α and YAP crosstalk was observed in endothelial cells. The role of the Hippo pathway and in particular YAP in endothelial cells has been recognised mostly during the process of blood vessel formation. Deletion of YAP in mice led to abnormal vascular development leading to alterations in retinal and brain vessels [74]. In contrast, YAP activation was found to be important as a positive regulator of angiogenesis via Signal transducer and activator of transcription 3 (STAT3) [75] or Vascular endothelial growth factor (VEGF) signalling [76].

In a more recent study by Choi et al. the role of YAP in endothelial cells was characterized in the setting of vascular inflammation [77]. During pathological conditions such as atherosclerosis or chronic hypertension, inflammatory response in the endothelial cells is very important. In these conditions, TNF-α is understood to be one of the central pro-inflammatory regulators. Interestingly, Choi and colleagues found that stimulation of Human vascular endothelial (HUVEC) cells with TNF-α induces YAP activity by reducing LATS1, TAZ and YAP phosphorylation [77]. This effect was dependent on RhoGTPase activity, which is consistent with previous findings showing associations between LATS1 phosphorylation, RhoGTPase and Rho associated coiled-coil containing protein kinase 1 (ROCK1) [78].The functional implication of TNF-α induced YAP activation in HUVEC seems to be associated with the induction of Vascular cell adhesion molecule 1 (VCAM1) and Intercellular adhesion molecule 1 (ICAM1) expression, which are important modulators of leukocyte adhesion to the endothelial cells [77]. Leukocyte adhesion is very important in the endothelial inflammatory response and is a crucial step during atherosclerotic plaque development. The inhibition of YAP or activation of core Hippo components might therefore become a novel approach to halt the process.

## 6. Concluding Remarks

**Figure 3:**
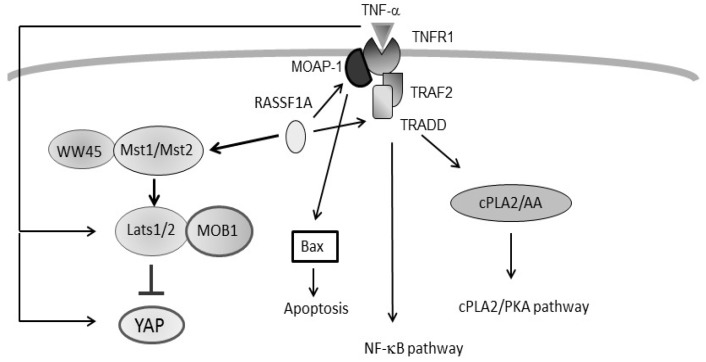
Schematic diagram of the interaction between TNF-α and the Hippo signalling pathway.

Both the TNF-α and the Hippo signalling pathways play major roles in the regulation of cell death and apoptosis. A large number of studies have characterized the upstream modulators and downstream effectors of the two pathways. It appears that several components of the Hippo pathway interact with and regulate the effectors of the TNF-α pathway. On the other hand, TNF-α itself is able to modulate the activation of LATS2 and YAP, two major components of the Hippo pathway (Figure 3). 

The close relationship between the TNF-α and the Hippo pathways may provide us with new insights into how to interfere with or modulate these pathways to produce beneficial effects. Further studies dissecting the cross-talk and intersection between these two signalling pathways are essential in order to completely understand the functional implications of the interactions, and how they can be manipulated for therapeutic purposes. 

## Abbreviations

TNF-α	Tumour necrosis factor-alpha
RASSF	Ras-association domain family member
TRAIL	TNF-related apoptosis-inducing ligand
DISC	Death inducing signalling complex
Bcl-2	B-cell lymphoma 2
Apaf-1	Apoptosis protease-activating factor 1
ERK	extracellular signal–regulated kinase
YAP	Yes-associated protein
TAZ	Tafazzin
TEAD	TEA domain family member
MST	Mammalian Ste20-like kinase
LATS	Large tumour suppressor
TACE	TNF-α converting enzyme
TNFR	TNF receptor; DD, death domain
TRAD	TNFR-associated death domain protein
TRAF	TNFR-associated factor
RIPK1	Receptor-interacting serine/threonine-protein kinase 1
FADD	Fas associated protein with death domain
NF-κB	Nuclear factor kappa-light-chain-enhancer of activated B cells
SODD	Silencer of death domain
MAPK	Mitogen-activated protein kinase
JNK	c-Jun-N-terminal kinase
IκB	inhibitor of kappa B
RIP	receptor interacting protein kinase
IKK	IκB kinase
5-HETE	5-hydroxyeicosatetraenoic acid
Yki	Yorkie
TAO1	Serine/threonine-protein kinase TAO1
SAV1	Salvador homolog
MOB1	MOB kinase activator 1
AJ	adherens junction
TJ	tight junction
LKB1	Liver kinase B1
NPHP4	Nephronophthisis 4
AMOT	angiomotin
ECM	Extracellular matrix
GPCR	G-protein coupled receptor
ZO	Zona occludens
LPA	Lysophosphatidic acid
MOAP1	Modulator of apoptosis 1
Bax	Bcl-2-associated x protein
SARAH	Salvador RASSF Hippo
cPLA2	phospholipase A2
TLR	toll-like receptor
MyD88	Myeloid differentiation primary response 88
IRAK	Interleukin-1 receptor-associated kinase
Bcl-xL	B-cell lymphoma-extra large
STAT3	Signal transducer and activator of transcription 3
VEGF	Vascular endothelial growth factor
HUVEC	Human vascular endothelial cell
ROCK1	Rho associated coiled-coil containing protein kinase 1
VCAM1	Vascular cell adhesion molecule 1
ICAM1	Intercellular adhesion molecule 1
PKC	protein kinase C
ATM	ataxia telangiectasia mutated
TAK1	Transforming growth factor beta-activated kinase 1
